# Supplementary material for: Genetic Diversity of Historical and Modern Populations of Russian Cattle Breeds Revealed by Microsatellite Analysis
Source: Genes (Basel). 2020 Aug 14;11(8):940. doi: 10.3390/genes11080940 (PMC7463645; doi:10.3390/genes11080940)
Supplement: Supplementary file 1 [file genes-11-00940-s001.zip › Suppl_Fig_1_foto.pdf]

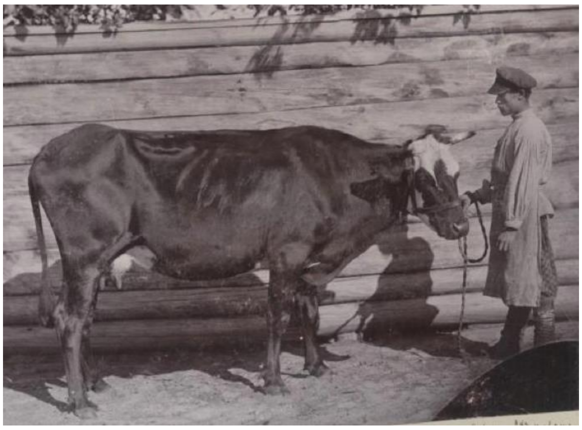

Cow of Kholmogor breed, year 1902[a]

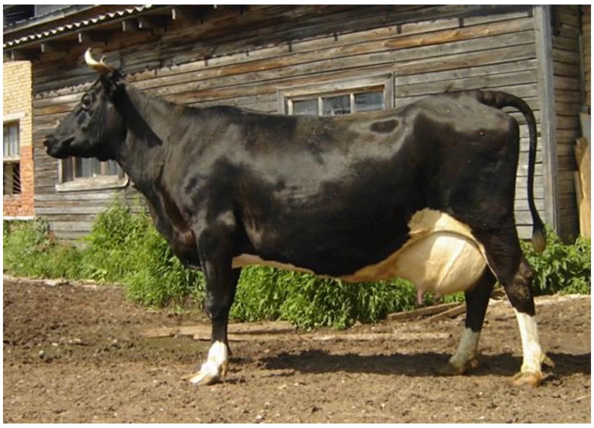

Modern cow of Kholmogor breed[c]

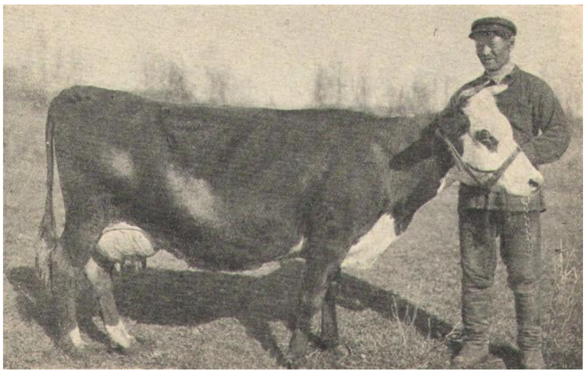

Cow of Yaroslavl breed, year 1925[b]

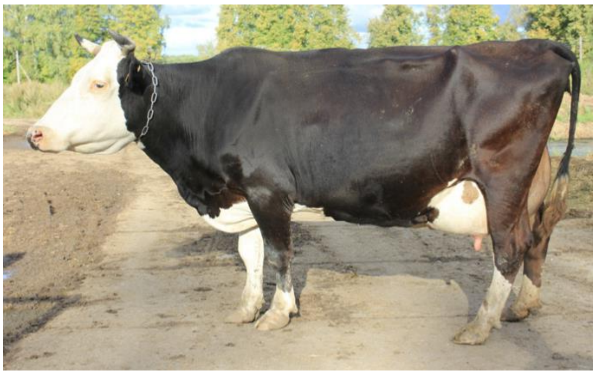

Modern cow of Yaroslavl breed[d]

Figure S1. Historical and modern representatives of Kholmogor and Yaroslavl breeds

Sources:

[a] Photo from the open sources of the "Tobolsk historical and architectural Museum-reserve", available online http://goskatalog.ru/portal/#/collections?id=3699723 (accessed 05.07.2020).

[b] The state pedigree book of cattle of Yaroslavl breed. People's Commissariat of agriculture of the RSFSR 1929, 3(2), 214 p. (in Russian)

[c] Matyukov V.S., Zharikov Ya.A., Zinovieva N.A. Genetic history and value of the gene pool of the disappearing Kholmogor breed. Dairy and meat cattle breeding, 2018, 2, 2-8 (in Russian)

[d] Catalog of AI bulls. JSC "Yarplem" 2018, 120 p. (in Russian)
