# Supplementary material for: Genetic Diversity of Historical and Modern Populations of Russian Cattle Breeds Revealed by Microsatellite Analysis
Source: Genes (Basel). 2020 Aug 14;11(8):940. doi: 10.3390/genes11080940 (PMC7463645; doi:10.3390/genes11080940)
Supplement: Supplementary file 1 [file genes-11-00940-s001.zip › Suppl_Tab_S1.pdf]

#	Sample ID	Breed	Qubit, ng/mcl	OD260/OD280	No replicates	TGLA227							
						A1	A2	+PCR	ADO	FA	Err	QI	A1
1	H1	KH	26.0	1.97	5	81	89	5	0	0	0	1.00	125
2	H2	KH	47.6	1.84	5	81	89	5	0	0	0	1.00	125
3	H3	KH	10.9	1.95	5	81	81	5	0	0	0	1.00	135
4	H5	KH	18.4	1.97	5	83	83	5	0	0	0	1.00	139
5	H6	KH	2.94	1.96	5	81	93	5	0	0	0	1.00	137
6	H7	KH	8.14	1.81	5	81	81	5	0	0	0	1.00	125
7	H8	KH	1.17	1.74	5	89	91	5	0	0	0	1.00	125
8	H9	KH	9.00	1.92	5	89	91	5	0	0	0	1.00	139
9	H12	KH	1.97	1.66	5	81	83	5	0	0	0	1.00	125
10	H14	KH	11.9	1.92	5	93	97	5	0	0	0	1.00	135
11	H16	KH	25.8	2.00	5	81	89	5	0	0	0	1.00	137
12	H17	KH	6.56	1.95	5	83	89	5	0	0	0	1.00	127
13	H18	KH	14.9	2.00	5	81	89	5	0	0	0	1.00	125
14	H19	KH	13.6	1.87	5	81	101	5	0	1	1	0.80	137
15	H20	KH	6.18	1.88	5	81	89	5	0	0	0	1.00	135
16	H21	KH	10.0	1.92	5	89	91	5	0	0	0	1.00	127
17	H22	KH	20.0	1.88	5	81	83	5	0	0	0	1.00	125
18	H24	KH	3.30	1.82	5	81	83	5	0	0	0	1.00	137
19	H25	KH	63.4	1.96	5	87	91	5	0	0	0	1.00	137
20	H27	KH	9.98	1.73	5	89	97	5	0	0	0	1.00	127
21	H29	KH	6.70	1.85	5	89	93	5	0	0	0	1.00	137
22	H30	KH	3.18	1.70	5	81	89	5	0	0	0	1.00	127
23	H33	YR	6.58	1.82	5	77	91	5	0	0	0	1.00	127
24	H35	YR	8.82	1.83	8	101	103	8	0	0	0	1.00	127
25	H36	YR	13.6	1.88	8	83	91	8	0	0	0	1.00	125
26	H37	YR	11.7	1.88	5	83	93	5	0	0	0	1.00	125
27	H39	YR	8.08	1.85	8	83	87	8	0	0	0	1.00	121
28	H40	YR	1.04	1.85	8	81	93	8	0	0	0	1.00	127
29	H41	YR	9.44	1.92	5	81	81	5	0	0	0	1.00	135
30	H42	YR	8.78	1.74	5	83	93	5	0	1	1	0.80	135
31	H43	YR	1.88	1.82	5	89	91	5	0	0	0	1.00	125
32	H44	YR	8.68	1.98	8	81	93	8	0	0	0	1.00	137
33	H46	YR	1.23	1.69	5	83	87	5	0	0	0	1.00	125
34	H47	YR	1.98	1.93	5	91	91	5	0	0	0	1.00	135
35	H50	YR	1.97	1.74	5	79	81	5	0	0	0	1.00	125
36	H51	YR	35.4	1.93	5	89	93	4	0	0	0	1.00	125
37	H52	YR	7.76	1.85	5	81	99	5	0	0	0	1.00	131
38	H55	YR	6.64	1.86	5	83	87	5	0	0	0	1.00	135
39	H56	YR	17.0	1.97	5	87	91	5	1	0	1	0.80	135
40	H62	YR	5.16	1.77	5	91	101	5	0	0	0	1.00	125
41	H63	YR	1.59	1.73	5	81	87	5	0	0	0	1.00	135
42	H67	YR	2.94	1.85	5	81	97	5	0	0	0	1.00	125
43	H71	GR	9.42	1.88	5	79	97	5	0	0	0	1.00	129
44	H72	GR	4.14	1.93	5	89	91	5	0	0	0	1.00	135
45	H99	NV	5.26	1.86	8	97	97	8	0	1	1	0.88	133
46	H101	NV	9.16	1.82	5	89	93	5	0	0	0	1.00	127
47	H111	HL	7.12	1.76	8	87	87	7	1	0	1	0.86	127
48	H112	HL	6.5	1.83	8	89	89	8	0	0	0	1.00	137

49	H113	HL	4.78	1.66	8	**89**	**93**	8	0	0	0	1.00	**133**

Sample ID - individual number of sample

Qubit - concentration of double-stranded DNA (ng/mcl)

OD260/OD280 - OD ratio

+PCR - number of positive PCRs

ADO - allelic drop-out

FA - false allele

Err - error

QI - quality indices

BM2113						ETH10							SPS11			
A2	+PCR	ADO	FA	Err	QI	A1	A2	+PCR	ADO	FA	Err	QI	A1	A2	+PCR	ADO
137	5	0	0	0	1.00	219	221	5	0	0	0	1.00	248	252	5	0
139	5	0	0	0	1.00	217	219	5	0	0	0	1.00	248	248	5	0
139	5	0	0	0	1.00	213	219	5	0	0	0	1.00	252	256	5	0
139	5	0	0	0	1.00	217	219	5	0	0	0	1.00	248	252	5	0
139	5	0	0	0	1.00	219	219	5	0	0	0	1.00	248	248	5	0
137	5	0	0	0	1.00	219	219	5	0	0	0	1.00	248	252	5	0
137	5	0	0	0	1.00	213	219	5	0	0	0	1.00	248	254	5	0
141	5	0	0	0	1.00	219	221	5	0	0	0	1.00	248	256	5	0
125	5	0	0	0	1.00	217	221	5	0	0	0	1.00	248	252	5	0
137	5	0	0	0	1.00	217	219	5	0	0	0	1.00	248	256	5	0
137	5	0	0	0	1.00	213	219	5	1	0	1	0.80	248	252	5	0
139	5	0	0	0	1.00	219	221	5	0	0	0	1.00	248	248	5	0
125	5	0	0	0	1.00	217	219	5	0	0	0	1.00	248	252	5	0
139	5	0	0	0	1.00	219	219	5	0	0	0	1.00	248	254	5	0
137	5	0	0	0	1.00	213	213	5	0	0	0	1.00	248	256	5	0
137	5	0	0	0	1.00	219	219	5	0	0	0	1.00	248	248	5	0
133	5	0	0	0	1.00	223	225	5	0	0	0	1.00	248	248	5	0
139	5	0	0	0	1.00	213	219	5	0	0	0	1.00	248	256	5	0
139	5	0	0	0	1.00	217	219	5	0	0	0	1.00	248	248	5	0
139	5	0	0	0	1.00	219	221	5	0	0	0	1.00	252	256	5	0
139	5	0	0	0	1.00	213	219	5	0	0	0	1.00	248	248	5	0
133	5	0	0	0	1.00	219	219	5	0	0	0	1.00	252	254	5	0
135	5	0	0	0	1.00	217	219	5	0	0	0	1.00	254	260	5	0
135	8	0	0	0	1.00	219	223	8	0	0	0	1.00	248	248	8	2
139	8	0	0	0	1.00	219	221	8	1	1	2	0.75	248	260	8	1
139	5	0	0	0	1.00	221	223	5	0	0	0	1.00	248	250	5	0
141	8	0	1	1	0.88	217	219	6	0	0	0	1.00	248	256	7	1
139	8	0	0	0	1.00	213	219	8	2	0	2	0.75	248	248	5	0
137	5	1	0	1	0.80	219	219	5	0	0	0	1.00	248	248	5	0
139	5	0	0	0	1.00	221	221	4	0	0	0	1.00	248	252	5	0
135	5	0	0	0	1.00	221	223	5	0	0	0	1.00	248	256	5	1
139	8	0	0	0	1.00	215	221	6	1	0	1	0.83	248	248	7	0
135	5	0	0	0	1.00	219	219	5	0	0	0	1.00	248	256	4	0
137	5	0	0	0	1.00	217	217	5	0	0	0	1.00	248	260	5	0
125	5	0	0	0	1.00	217	217	5	0	0	0	1.00	252	260	5	0
135	5	0	0	0	1.00	217	219	5	0	0	0	1.00	248	260	5	0
139	5	0	0	0	1.00	217	221	5	0	0	0	1.00	248	252	5	0
137	5	0	0	0	1.00	217	223	5	0	0	0	1.00	252	254	5	0
141	5	0	0	0	1.00	219	219	5	0	0	0	1.00	248	248	5	0
125	5	0	0	0	1.00	217	219	5	0	0	0	1.00	248	256	5	0
137	5	0	0	0	1.00	217	219	5	0	0	0	1.00	248	252	5	0
125	5	0	0	0	1.00	219	221	5	0	0	0	1.00	248	248	5	0
139	5	0	0	0	1.00	217	225	5	0	0	0	1.00	248	254	5	0
135	5	0	0	0	1.00	219	219	5	0	0	0	1.00	248	254	5	0
139	8	0	0	0	1.00	219	219	8	0	0	0	1.00	248	252	8	2
129	5	0	0	0	1.00	211	219	5	0	0	0	1.00	250	254	5	0
139	8	2	0	2	0.75	217	219	7	1	0	1	0.86	248	248	5	0
139	8	0	0	0	1.00	219	219	6	0	0	0	1.00	248	248	5	0

**135**	8	0	0	0	1.00	**215**	**217**	8	0	0	0	1.00	**248**	**248**	5	0

5			TGLA122							INRA23						
FA	Err	QI	**A1**	**A2**	+PCR	ADO	FA	Err	QI	**A1**	**A2**	+PCR	ADO	FA	Err	QI
0	0	1.00	**143**	**153**	5	0	0	0	1.00	**206**	**210**	5	0	0	0	1.00
0	0	1.00	**143**	**143**	5	0	0	0	1.00	**206**	**214**	5	0	0	0	1.00
0	0	1.00	**143**	**143**	5	0	0	0	1.00	**198**	**206**	5	0	0	0	1.00
0	0	1.00	**143**	**153**	5	0	0	0	1.00	**210**	**214**	5	0	0	0	1.00
0	0	1.00	**151**	**171**	5	0	0	0	1.00	**206**	**208**	5	0	0	0	1.00
0	0	1.00	**143**	**147**	5	0	0	0	1.00	**206**	**216**	5	0	0	0	1.00
0	0	1.00	**143**	**169**	5	0	0	0	1.00	**208**	**214**	5	0	0	0	1.00
0	0	1.00	**143**	**151**	5	0	0	0	1.00	**208**	**212**	5	0	0	0	1.00
0	0	1.00	**143**	**179**	5	0	0	0	1.00	**208**	**212**	5	0	0	0	1.00
0	0	1.00	**141**	**143**	5	0	0	0	1.00	**214**	**214**	5	0	0	0	1.00
0	0	1.00	**143**	**161**	5	0	1	1	0.80	**206**	**210**	5	0	0	0	1.00
0	0	1.00	**141**	**151**	5	0	0	0	1.00	**210**	**212**	5	0	0	0	1.00
0	0	1.00	**143**	**143**	5	0	0	0	1.00	**208**	**212**	5	0	0	0	1.00
0	0	1.00	**143**	**151**	5	0	0	0	1.00	**198**	**212**	5	0	0	0	1.00
0	0	1.00	**143**	**143**	5	0	0	0	1.00	**208**	**216**	5	0	0	0	1.00
0	0	1.00	**143**	**163**	5	0	0	0	1.00	**206**	**214**	5	0	0	0	1.00
0	0	1.00	**151**	**177**	5	0	0	0	1.00	**206**	**214**	5	0	0	0	1.00
0	0	1.00	**141**	**143**	5	0	0	0	1.00	**214**	**216**	5	0	0	0	1.00
0	0	1.00	**143**	**143**	5	0	0	0	1.00	**208**	**210**	5	0	0	0	1.00
0	0	1.00	**143**	**149**	5	0	0	0	1.00	**212**	**214**	5	0	0	0	1.00
0	0	1.00	**143**	**153**	5	0	0	0	1.00	**214**	**214**	5	0	0	0	1.00
0	0	1.00	**143**	**151**	5	0	0	0	1.00	**198**	**210**	5	0	0	0	1.00
0	0	1.00	**143**	**143**	5	0	0	0	1.00	**206**	**214**	5	0	0	0	1.00
0	2	0.75	**143**	**153**	8	0	0	0	1.00	**208**	**212**	8	1	1	2	0.75
0	1	0.88	**141**	**153**	8	0	0	0	1.00	**206**	**214**	8	1	0	1	0.88
0	0	1.00	**143**	**151**	5	0	0	0	1.00	**206**	**214**	5	0	0	0	1.00
0	1	0.86	**143**	**143**	8	0	0	0	1.00	**204**	**208**	7	1	0	1	0.86
1	1	0.80	**143**	**167**	8	0	0	0	1.00	**208**	**214**	5	0	0	0	1.00
0	0	1.00	**143**	**143**	5	0	0	0	1.00	**202**	**214**	5	0	0	0	1.00
0	0	1.00	**151**	**153**	5	0	0	0	1.00	**214**	**214**	5	0	0	0	1.00
0	1	0.80	**143**	**143**	5	0	1	1	0.80	**206**	**214**	5	0	0	0	1.00
0	0	1.00	**143**	**149**	8	1	0	1	0.88	**204**	**214**	5	0	0	0	1.00
0	0	1.00	**143**	**151**	5	0	0	0	1.00	**208**	**214**	4	1	0	1	0.75
0	0	1.00	**151**	**151**	5	0	0	0	1.00	**210**	**214**	5	0	0	0	1.00
0	0	1.00	**151**	**157**	5	0	0	0	1.00	**208**	**208**	5	0	0	0	1.00
0	0	1.00	**141**	**147**	5	0	0	0	1.00	**198**	**206**	5	0	0	0	1.00
0	0	1.00	**151**	**169**	5	0	0	0	1.00	**212**	**214**	5	0	0	0	1.00
0	0	1.00	**151**	**153**	5	0	0	0	1.00	**206**	**214**	5	0	0	0	1.00
0	0	1.00	**141**	**181**	5	1	0	1	0.80	**206**	**214**	**5**	1	0	1	0.80
0	0	1.00	**151**	**163**	5	0	0	0	1.00	**212**	**214**	5	0	0	0	1.00
0	0	1.00	**151**	**153**	5	0	0	0	1.00	**202**	**206**	5	0	0	0	1.00
0	0	1.00	**143**	**161**	5	0	0	0	1.00	**202**	**206**	5	0	0	0	1.00
0	0	1.00	**153**	**153**	5	0	0	0	1.00	**198**	**206**	5	0	0	0	1.00
0	0	1.00	**139**	**139**	5	0	0	0	1.00	**198**	**212**	5	0	0	0	1.00
0	2	0.75	**143**	**151**	8	0	0	0	1.00	**214**	**214**	8	0	1	1	0.88
1	1	0.80	**143**	**151**	5	0	0	0	1.00	**206**	**212**	5	0	1	1	0.80
0	0	1.00	**143**	**143**	6	0	1	1	0.83	**206**	**206**	6	0	0	0	1.00
0	0	1.00	**151**	**161**	8	2	0	2	0.75	**206**	**206**	4	0	0	0	1.00

1	1	0.80	**143**	**161**	8	0	0	0	1.00	**208**	**210**	6	0	0	0	1.00

TGLA126							ETH225							B		
**A1**	**A2**	+PCR	ADO	FA	Err	QI	**A1**	**A2**	+PCR	ADO	FA	Err	QI	**A1**	**A2**	+PCR
**119**	**123**	5	0	0	0	1.00	**150**	**150**	5	0	0	0	1.00	**178**	**182**	5
**115**	**117**	5	0	0	0	1.00	**148**	**150**	5	0	0	0	1.00	**180**	**182**	5
**115**	**117**	5	0	0	0	1.00	**150**	**150**	5	0	0	0	1.00	**178**	**182**	5
**117**	**119**	5	1	0	1	0.80	**148**	**150**	5	0	0	0	1.00	**180**	**182**	5
**119**	**123**	5	0	0	0	1.00	**148**	**150**	5	0	0	0	1.00	**180**	**182**	5
**115**	**117**	5	0	0	0	1.00	**150**	**150**	5	0	0	0	1.00	**188**	**188**	5
**117**	**117**	5	0	0	0	1.00	**140**	**148**	5	0	0	0	1.00	**178**	**188**	5
**115**	**123**	5	0	0	0	1.00	**150**	**150**	5	0	0	0	1.00	**180**	**182**	5
**117**	**123**	5	0	0	0	1.00	**144**	**148**	5	0	0	0	1.00	**182**	**182**	5
**117**	**119**	5	0	0	0	1.00	**150**	**150**	5	0	0	0	1.00	**178**	**182**	5
**117**	**123**	5	0	0	0	1.00	**144**	**148**	5	0	0	0	1.00	**178**	**182**	5
**115**	**123**	5	0	0	0	1.00	**148**	**150**	5	0	0	0	1.00	**178**	**182**	5
**117**	**123**	5	1	0	1	0.80	**144**	**148**	5	0	0	0	1.00	**182**	**188**	5
**115**	**115**	5	0	0	0	1.00	**144**	**144**	5	0	0	0	1.00	**180**	**180**	5
**117**	**117**	5	0	0	0	1.00	**140**	**152**	5	0	0	0	1.00	**178**	**182**	5
**115**	**117**	5	0	0	0	1.00	**144**	**150**	5	0	0	0	1.00	**182**	**188**	5
**115**	**123**	5	1	0	1	0.80	**144**	**150**	5	0	0	0	1.00	**178**	**188**	5
**115**	**123**	5	1	0	1	0.80	**148**	**148**	5	0	0	0	1.00	**182**	**182**	5
**117**	**123**	5	0	0	0	1.00	**144**	**150**	5	0	0	0	1.00	**178**	**180**	5
**115**	**117**	5	0	0	0	1.00	**144**	**148**	5	0	0	0	1.00	**178**	**182**	5
**117**	**123**	5	0	0	0	1.00	**148**	**150**	5	0	0	0	1.00	**180**	**188**	5
**115**	**117**	5	0	0	0	1.00	**150**	**150**	5	0	0	0	1.00	**178**	**178**	5
**117**	**121**	5	0	0	0	1.00	**146**	**150**	5	0	0	0	1.00	**188**	**188**	5
**115**	**117**	8	0	0	0	1.00	**144**	**150**	8	0	0	0	1.00	**178**	**180**	8
**117**	**123**	8	0	0	0	1.00	**146**	**150**	8	0	0	0	1.00	**180**	**182**	8
**115**	**117**	5	0	0	0	1.00	**150**	**152**	5	0	0	0	1.00	**178**	**182**	5
**117**	**125**	8	1	0	1	0.88	**144**	**150**	8	0	0	0	1.00	**180**	**188**	8
**115**	**115**	8	0	1	1	0.88	**142**	**148**	8	0	0	0	1.00	**178**	**180**	8
**115**	**117**	5	0	0	0	1.00	**144**	**150**	5	0	0	0	1.00	**178**	**178**	5
**115**	**117**	5	0	0	0	1.00	**146**	**150**	4	0	0	0	1.00	**182**	**182**	5
**117**	**123**	5	0	0	0	1.00	**140**	**146**	5	0	0	0	1.00	**178**	**188**	5
**115**	**119**	8	1	1	2	0.75	**148**	**150**	6	0	0	0	1.00	**182**	**182**	7
**117**	**121**	5	0	0	0	1.00	**144**	**148**	5	0	0	0	1.00	**188**	**188**	5
**117**	**117**	5	0	0	0	1.00	**146**	**150**	5	0	0	0	1.00	**182**	**182**	5
**117**	**123**	5	0	0	0	1.00	**140**	**146**	5	0	0	0	1.00	**180**	**188**	5
**117**	**117**	5	0	0	0	1.00	**144**	**146**	5	0	0	0	1.00	**180**	**180**	5
**115**	**117**	5	0	0	0	1.00	**144**	**150**	5	0	0	0	1.00	**178**	**182**	5
**117**	**119**	5	0	0	0	1.00	**146**	**150**	5	0	0	0	1.00	**178**	**182**	5
**121**	**123**	5	1	0	1	0.80	**150**	**150**	5	0	0	0	1.00	**178**	**180**	5
**115**	**117**	5	0	0	0	1.00	**148**	**148**	5	0	0	0	1.00	**178**	**182**	5
**115**	**115**	5	0	0	0	1.00	**144**	**150**	5	0	0	0	1.00	**178**	**180**	5
**115**	**121**	5	1	0	1	0.80	**146**	**150**	5	0	0	0	1.00	**180**	**180**	5
**115**	**117**	5	0	0	0	1.00	**148**	**160**	5	0	0	0	1.00	**182**	**188**	5
**121**	**123**	5	0	0	0	1.00	**140**	**144**	5	0	0	0	1.00	**180**	**180**	5
**123**	**123**	5	0	0	0	1.00	**150**	**150**	6	0	1	1	0.83	**180**	**180**	8
**115**	**127**	5	0	0	0	1.00	**148**	**152**	5	0	0	0	1.00	**180**	**180**	5
**121**	**121**	5	0	0	0	1.00	**148**	**148**	8	0	0	0	1.00	**182**	**188**	8
**115**	**117**	8	0	0	0	1.00	**148**	**150**	8	2	0	2	0.75	**180**	**182**	8

**117**	**125**	7	0	0	0	1.00	**148**	**148**	8	0	0	0	1.00	**180**	**184**	7

M1824			
ADO	FA	Err	QI
0	0	0	1.00
0	0	0	1.00
0	0	0	1.00
0	0	0	1.00
0	0	0	1.00
0	0	0	1.00
0	0	0	1.00
0	0	0	1.00
0	0	0	1.00
0	0	0	1.00
0	0	0	1.00
0	0	0	1.00
0	0	0	1.00
0	0	0	1.00
0	0	0	1.00
0	0	0	1.00
0	0	0	1.00
0	0	0	1.00
0	0	0	1.00
0	0	0	1.00
0	0	0	1.00
0	0	0	1.00
0	0	0	1.00
1	0	1	0.88
0	0	0	1.00
0	0	0	1.00
0	0	0	1.00
2	0	2	0.75
0	0	0	1.00
0	0	0	1.00
1	0	1	0.80
0	1	1	0.86
0	0	0	1.00
0	0	0	1.00
0	1	1	0.80
0	0	0	1.00
0	0	0	1.00
0	0	0	1.00
0	0	0	1.00
0	0	0	1.00
0	0	0	1.00
0	0	0	1.00
0	0	0	1.00
0	0	0	1.00
0	0	0	1.00
0	0	0	1.00
2	0	2	0.75
0	0	0	1.00

0	0	0	1.00
