# Supplementary material for: Genetic Diversity of Historical and Modern Populations of Russian Cattle Breeds Revealed by Microsatellite Analysis
Source: Genes (Basel). 2020 Aug 14;11(8):940. doi: 10.3390/genes11080940 (PMC7463645; doi:10.3390/genes11080940)
Supplement: Supplementary file 1 [file genes-11-00940-s001.zip › Suppl_Tab_S2.pdf]

Table S2. Quality of genotyping at different loci based on quality indices (QIs)[1]

#	Locus	Mean QI value (M ± SE)	QI = 1		0.75 ≤ QI < 1.00		Probability (P) of correct genotyping[2]
			Number of samples, n	Ratio of samples, %	Number of samples, n	Ratio of samples, %	
1	BM2113	0.988±0.007	46	93.9	3	6.1	***
2	BM1824	0.976±0.010	43	87.8	6	12.2	***
3	ETH10	0.979±0.009	41	83.7	8	16.3	***
4	ETH225	0.991±0.006	47	95.9	2	4.1	***
5	INRA023	0.974±0.010	42	85.7	7	14.3	***
6	SPS115	0.968±0.011	44	89.8	5	11.2	***
7	TGLA122	0.977±0.009	43	87.8	6	12.2	***
8	TGLA126	0.965±0.011	40	81.6	9	18.4	***
9	TGLA227	0.982±0.008	44	89.8	5	11.2	***

[1] Calculation of quality indices is describes in paragraph 2.3.

[2] Probability values of correct genotyping were estimated according He G. et al. [52]

*** P<0.001
