# Supplementary material for: Genetic Diversity of Historical and Modern Populations of Russian Cattle Breeds Revealed by Microsatellite Analysis
Source: Genes (Basel). 2020 Aug 14;11(8):940. doi: 10.3390/genes11080940 (PMC7463645; doi:10.3390/genes11080940)
Supplement: Supplementary file 1 [file genes-11-00940-s001.zip › Suppl_Tab_S3.pdf]

Table S3. Frequency of lost and newly emerged alleles in the studied historical and modern cattle populations*

Locus	Allele	Historical populations					Modern populations	
		KH_H	YR_H	GR_H	NV_H	HL_H	KH_M	YR_M
BM2113	121	-	0,025	-	-	-	-	-
	129	-	-	0,250	0,250	-	-	-
	131	-	0,025	-	-	-	-	-
BM1824	184	-	-	-	-	0,167	-	-
	190	-	-	-	-	-	0,003	0,082
ETH10	211	-	-	-	0,250	-	-	-
ETH225	158	-	-	-	-	-	-	0,008
	160	-	-	0,250	-	-	-	-
SPS115	258	-	-	-	-	-	-	0,016
INRA23	200	-	-	-	-	-	0,006	-
	204	-	0,050	-	-	-	-	-
	220	-	-	-	-	-	0,006	-
TGLA122	157	-	0,025	-	-	-	-	-
	167	-	0,025	-	-	-	-	-
	169	0,023	0,025	-	-	-	-	-
	173	-	-	-	-	-	0,054	0,205
	177	0,023	-	-	-	-	-	-
	179	0,023	-	-	-	-	-	-
	181	-	0,025	-	-	-	-	-
	183	-	-	-	-	-	-	0,008
TGLA126	111	-	-	-	-	-	-	0,016
	113	-	-	-	-	-	-	0,025
	125	-	0,025	-	-	0,167	-	-
	127	-	-	-	0,250	-	-	-
TGLA227	75	-	-	-	-	-	0,006	0,057
	85	-	-	-	-	-	-	0,008
	95	-	-	-	-	-	0,164	-

*Modern Holsteins were excluded from the analysis; studied historical populations: KH_H – Kholmogor breed, YR_H – Yaroslavl breed, GR_H – Great Russian cattle, NV_H – Novgorod cattle, HL_H – Holland cattle; modern populations: KH_M – Kholmogor breed, YR_M – Yaroslavl breed; emerged alleles are presented above the bold line; Lost alleles are presented below the bold line.
