# Supplementary material for: Genetic Diversity of Historical and Modern Populations of Russian Cattle Breeds Revealed by Microsatellite Analysis
Source: Genes (Basel). 2020 Aug 14;11(8):940. doi: 10.3390/genes11080940 (PMC7463645; doi:10.3390/genes11080940)
Supplement: Supplementary file 1 [file genes-11-00940-s001.zip › Suppl_Tab_S4.pdf]

Table S4. Distribution of allelic drop-outs for the shorter and the longer allele in heterozygous genotypes.

#	Locus	Average length, bp	Number of allelic drop-outs		X^2 test	P value
			Longer allele	Shorter allele		
1	BM2113	133,3	2	1	0,333	0,564
2	BM1824	181,5	3	3	0,000	ns
3	ETH10	218,5	1	5	2,667	0,103
4	ETH225	147,7	2	0	2,000	0,157
5	INRA023	209,2	4	1	1,800	0,180
6	SPS115	250,6	5	2	1,286	0,257
7	TGLA122	149,0	4	0	4,000*	0,046
8	TGLA126	118,2	7	1	4,500*	0,034
9	TGLA227	87,7	2	0	2,000	0,157

* $P < 0.05$
