# Supplementary figures and images for: Genetic Diversity of Historical and Modern Populations of Russian Cattle Breeds Revealed by Microsatellite Analysis
Source: Genes (Basel). 2020 Aug 14;11(8):940. doi: 10.3390/genes11080940 (PMC7463645; doi:10.3390/genes11080940)

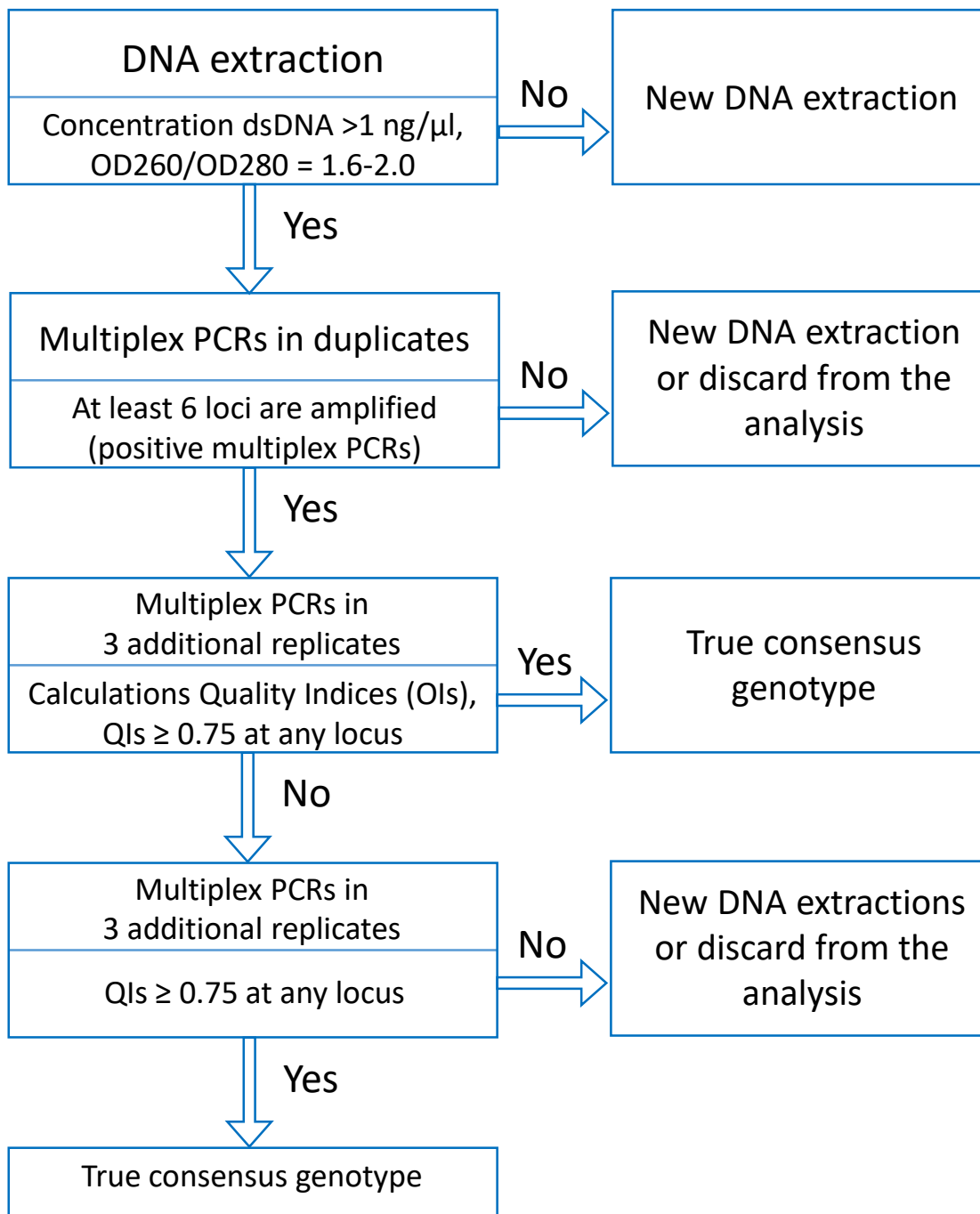

Supplement: Supplementary file 1 [file genes-11-00940-s001.zip › Suppl_Fig_2_scheme.pdf]

$$\text{Delta K} = \text{mean}(|L''(K)|) / \text{stdev}[L(K)]$$

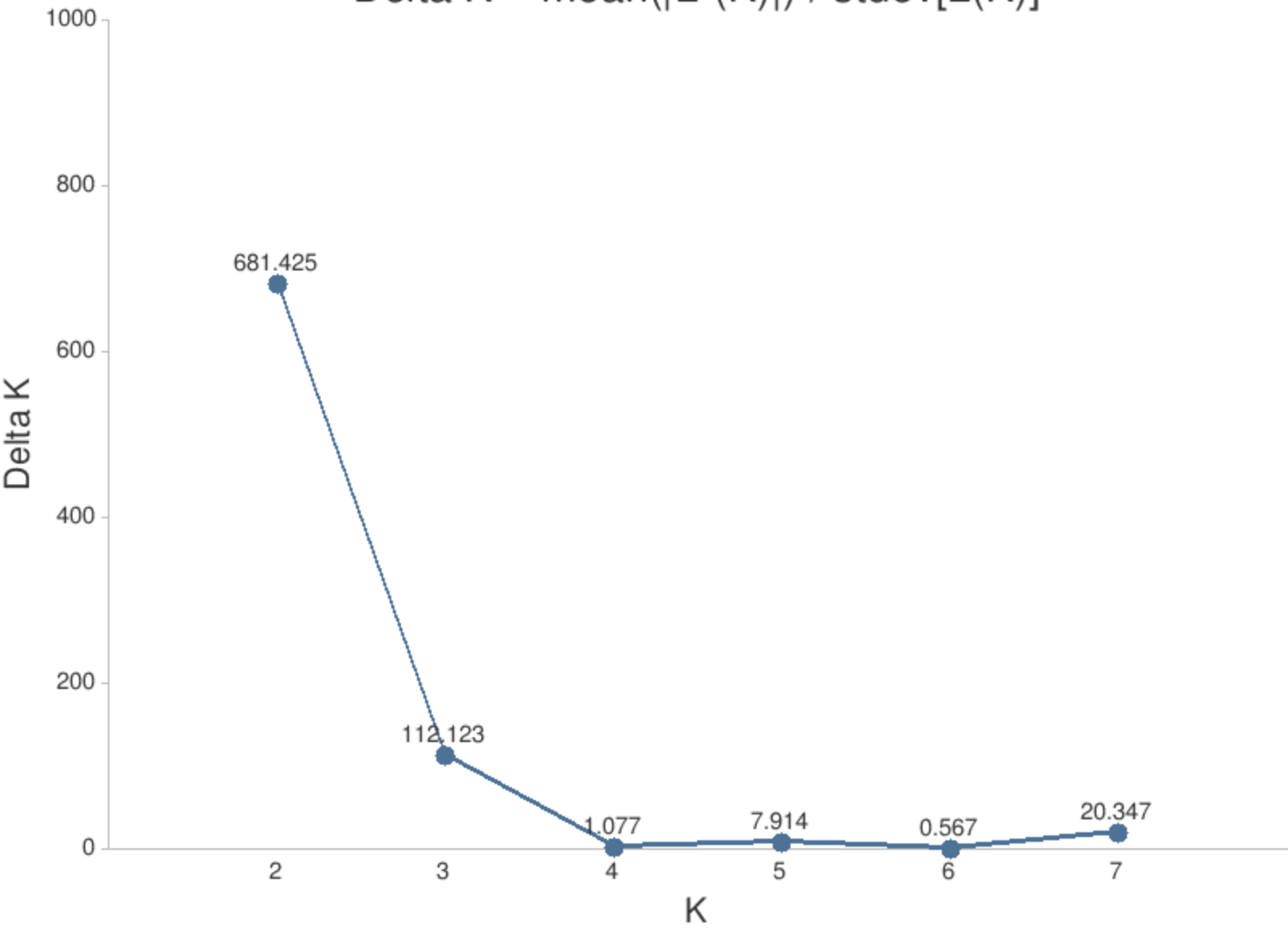

Supplement: Supplementary file 1 [file genes-11-00940-s001.zip › Suppl_Fig_3_K.pdf]
